# Characteristics of behavioral symptoms in right-sided predominant semantic dementia and their impact on caregiver burden: a cross-sectional study

**DOI:** 10.1186/s13195-021-00908-2

**Published:** 2021-10-09

**Authors:** Shunsuke Sato, Mamoru Hashimoto, Kenji Yoshiyama, Hideki Kanemoto, Maki Hotta, Shingo Azuma, Takashi Suehiro, Kyosuke Kakeda, Yoshitaka Nakatani, Sumiyo Umeda, Ryuji Fukuhara, Minoru Takebayashi, Manabu Ikeda

**Affiliations:** 1 Department of Psychiatry, Osaka University Graduate School of Medicine, D3, 2-2 Yamadaoka,, Suita City,, Osaka 565-0871 Japan; 2 Department of Psychiatry, Osaka General Medical Center, Osaka, Japan; 3 Department of Neuropsychiatry, Faculty of Life Sciences, Kumamoto University, Kumamoto, Japan; 4 Department of Psychiatry, Mizuma Hospital, Kaizuka, Japan; 5 Department of Psychiatry, Osaka Psychiatric Medical Center, Osaka, Japan; 6 Department of Psychiatry, Daini Osaka Police Hospital, Osaka, Japan; 7 Department of Neuropsychiatry, Kumamoto University Hospital, Kumamoto, Japan

**Keywords:** Frontotemporal dementia, Semantic dementia, Progressive non-fluent aphasia, Frontotemporal lobar degeneration, Primary progressive aphasia, Alzheimer’s disease, Dementia, Right temporal lobe atrophy, Behavioral symptoms, Caregiver burden

## Abstract

**Background:**

This study aimed to clarify the neuropsychiatric symptoms of right-sided predominant semantic dementia (SD-R) by comparing them with those of behavioral variant frontotemporal dementia (bvFTD), left-sided predominant SD (SD-L), and Alzheimer’s disease (AD). This study also aimed to identify clinical factors related to caregiver burden for bvFTD, SD-R, and SD-L.

**Methods:**

The neuropsychiatric symptoms of 28 patients with bvFTD, 14 patients with SD-R, 24 patients with SD-L, and 43 patients with AD were evaluated using the Neuropsychiatric Inventory (NPI) and the Stereotypy Rating Inventory (SRI). Cognitive function was assessed using the Mini-Mental State Examination (MMSE). Dementia severity was assessed using the Clinical Dementia Rating. Activities of daily living were assessed using the Lawton Instrument Activities of Daily Living (IADL) scale and the Physical Self-Maintenance Scale. We compared the NPI and SRI scores among the four groups using the Kruskal-Wallis test. In addition, clinical factors related to caregiver burden, represented by the Japanese version of the Zarit Burden Interview (J-ZBI), were analyzed using multiple regression analysis in the bvFTD, SD-R, and SD-L groups.

**Results:**

The NPI total score and the NPI subscale scores of apathy and disinhibition were significantly higher in the bvFTD group than in the SD-L and AD groups. The SD-R group scores were closer to those of the bvFTD group than the SD-L group. The SRI total score and SRI subscale scores for eating and cooking and speaking were significantly higher in the bvFTD, SD-R, and SD-L groups than in the AD group. The NPI total score was significantly associated with the J-ZBI score in the bvFTD group. The NPI total score and Lawton IADL scale score were independently associated with the J-ZBI score in the SD-R group. Furthermore, the NPI total score and MMSE score were independently associated with the J-ZBI score in the SD-L group.

**Conclusions:**

SD-R seemed to be a similar condition to bvFTD rather than SD-L regarding behavioral symptoms. Our results suggest that each frontotemporal dementia subgroup requires different approaches to reduce the caregiver burden.

## Background

Frontotemporal dementia (FTD) is a neurodegenerative disorder characterized by progressive behavioral disorders and/or language disability caused by atrophy and neuronal loss in the frontal and temporal lobes [1–3]. FTD includes three clinical subtypes: behavioral variant frontotemporal dementia (bvFTD), semantic dementia (SD), and progressive non-fluent aphasia (PNFA). In 2011, consensus clinical diagnostic criteria for primary progressive aphasia (PPA) were published, and SD and PNFA were classified as semantic variant PPA (svPPA) and non-fluent/agrammatic variant PPA, respectively [3]. When SD and PNFA were classified under the concept of PPA, there was a problem that behavioral disorders, which were an important feature of FTD, were not considered. Differences in neuropsychiatric symptoms among these subtypes have been reported [4–9]. Yiannopoulou et al. [9] reported that the total Neuropsychiatric Inventory (NPI) [10] score of bvFTD was significantly higher than that of both SD and PNFA. Park et al. [7] showed that bvFTD had a higher NPI score than PNFA. However, Rosen et al. [8] reported that both bvFTD and SD had a significantly higher NPI score than PNFA. These inconsistent results raise questions as to whether SD exhibit behavioral disorders comparable to bvFTD or whether they are milder than bvFTD.

A possible explanation for this discrepancy is that the effect of the laterality of brain atrophy in SD on neuropsychiatric symptoms has not been fully considered. SD is characterized by asymmetrical atrophy of the anterior temporal lobe [11]. Individuals with left-sided predominant SD (SD-L) exhibit severe language disturbance, whereas those with right-sided predominant SD (SD-R) exhibit prosopagnosia and behavioral changes from the early stage of the disease [1]. Despite having such different clinical profiles, patients with SD-L and SD-R have been analyzed without distinction in previous studies. In addition, because SD-R has a lower incidence and patients exhibit milder language disorders than SD-L [11, 12], SD-R might not have received much attention compared to SD-L. The poor understanding of the clinical symptoms of SD-R may also obscure the characteristics of behavioral symptoms in SD.

Recently, Ulugut Erkoyun et al. [5] reported detailed clinical-radiological findings of the right temporal variant of frontotemporal dementia (rtvFTD), a disease concept similar to SD-R. The clinical and neuropsychological characteristics of 70 patients with rtvFTD were compared with those of patients with svPPA, a concept similar to SD-L, bvFTD, and Alzheimer’s disease (AD). The authors found that prosopagnosia, episodic memory impairment, and behavioral changes such as disinhibition, apathy, compulsiveness, and loss of empathy were the most common initial symptoms in the rtvFTD group. In addition, distinctive symptoms of rtvFTD compared to the other groups included depression, somatic complaints, and motor/mental slowness. Their research has significantly improved our understanding of behavioral disorders of right-sided predominant SD, but little was mentioned about stereotypic behavior. Stereotypic behavior, which is a core symptom of bvFTD, can be as severe as bvFTD in SD [6]. Snowden et al. [13] reported that stereotypic behaviors were common in both bvFTD and SD and suggested that these behaviors had a more compulsive quality in SD. Josephs et al. [14] reported that patients with bvFTD with stereotypies had greater volume loss in the striatum compared to those without stereotypies and that the loss was more severe on the right, where it also extended laterally to the right insula. Therefore, the range and frequency of stereotypic behavior in SD, specifically in SD-R, are of potential interest.

Furthermore, the study by Ulugut Erkoyun et al. [5] did not assess caregiver burden for patients with rtvFTD. Caregiver burden is higher for caregivers of patients with bvFTD than for those with other forms of dementia because behavioral changes are usually the most distressing aspect of dementia for caregivers [15–17]. Thus, SD-R, in which changes in behavior are often the earliest symptom, may have a greater impact on caregivers than SD-L. However, the characteristics of caregiver burden in patients with SD are still incompletely understood.

This study aimed to clarify the characteristics of neuropsychiatric symptoms of SD-R, especially focusing on stereotypic behavior, by comparing them with those of bvFTD, SD-L, and AD. In addition, this study aimed to identify clinical factors related to caregiver burden for bvFTD, SD-R, and SD-L.

## Methods

### Participants

This retrospective observational study was conducted without intervention and in compliance with national legislation and the Declaration of Helsinki. This study was undertaken after obtaining approval from the Ethics Committee of Osaka University Medical Hospital and Kumamoto University Hospital.

This study included consecutive patients with bvFTD and SD who visited the dementia clinic of the Department of Psychiatry of Kumamoto University Hospital between April 2007 and March 2016 or attended the dementia clinic of the Department of Psychiatry of Osaka University Medical Hospital between April 2016 and August 2020. In addition, consecutive patients with AD who visited the dementia clinic of the Department of Psychiatry of Osaka University Medical Hospital between April 2016 and August 2020 were recruited as the control group. All patients were examined comprehensively by an experienced senior neuropsychiatrist (M.I.) and underwent routine laboratory tests, neuroimaging studies such as magnetic resonance imaging (MRI) and single-photon emission computed tomography, and standard neuropsychological examinations. Patients with bvFTD were diagnosed according to international consensus criteria for probable bvFTD [2]. AD was diagnosed according to the National Institute on Aging and Alzheimer’s Association criteria [18]. Patients with SD were diagnosed according to the Neary criteria [1]. In principle, PPA does not include patients who have prominent neuropsychiatric symptoms from the early stages of the disease. Thus, some SD-L patients with behavioral disorders from the early stages of the disease would be excluded from the SD group, strictly following the diagnostic criteria for PPA. On the other hand, when using the concept of rtvFTD as with Ulugut Erkoyun et al., patients with right temporal lobe atrophy without semantic memory impairment would be included. In this study, in order to clarify the difference between left-sided predominant and right-sided predominant semantic dementia, we first diagnosed SD using the Neary criteria [1]. Next, patients with SD were classified into two groups, SD-R and SD-L, based on the predominance of temporal lobe atrophy observed on MRI. The predominance of temporal lobe atrophy was determined by the side that had the higher medial temporal lobe atrophy score [5, 19], and we found no patients with equal atrophy of the bilateral temporal lobes. Left-handed and ambidextrous patients with SD were excluded from this study. Some patients with SD have behavioral changes comparable to those of bvFTD from the early stage of the disease. The initial behavioral changes in SD-R can make the differential diagnosis of bvFTD difficult. In this study, patients with both significant impairment on tests of semantic memory and temporal lobe dominant atrophy on MRI at the first visit were classified in the SD group, even if the patients developed behavioral disorders almost at the same time as semantic memory impairments. The following patients were excluded from this study: (1) those with an onset age of ≥76 years or older; (2) those with major psychiatric illness such as schizophrenia, major depression, or substance abuse; and (3) those without a reliable informant.

### Measures

We collected the patients’ demographic information, including estimated disease onset age, through clinical interviews with their caregivers. We defined disease onset age as the time when the patient’s closest caregiver became aware of their cognitive abnormality or behavioral symptoms. The patients’ behavioral and psychological statuses were assessed using the NPI [10]. The NPI evaluates ten neuropsychiatric disturbances common in dementia: delusions, hallucinations, agitation, depression, anxiety, euphoria, apathy, disinhibition, irritability, and aberrant motor behavior (AMB). Stereotypic behaviors were evaluated using the Stereotypy Rating Inventory (SRI) [20]. The SRI assesses five distinctive stereotypic behavioral disturbances often seen in patients with FTD: eating and cooking behaviors, roaming, speaking, movements, and daily rhythm. Eating and cooking behaviors include behavioral abnormalities such as eating and cooking the same dishes and buying the same foods they like. Roaming refers to a stereotypic walk, repeatedly going for a walk, taking the same route for a walk, or going to the same place. Speaking is a stereotyped speech, for example, telling the same story and repeatedly stating the same sentences and words. Movements are simple repetitive behaviors in which the patient makes the same movements, including rubbing their knees and clapping their hands repeatedly. Daily rhythm refers to a behavior in which the patient lives with a strictly fixed daily rhythm that looks like a timetable. Each subscale score of NPI and SRI is calculated by frequency (rated on a scale of 1–4) × severity (rated on a scale of 1–3), and the sum of all scores in each domain represents the NPI total score and the SRI total score.

Cognitive function was assessed using the Mini-Mental State Examination (MMSE) [21] and dementia severity using the Clinical Dementia Rating (CDR) [22]. The Lawton Instrument Activities of Daily Living (IADL) scale and the Physical Self-Maintenance Scale (PSMS) were used to evaluate activities of daily living [23, 24]. The Lawton IADL scale, which includes the ability to use the telephone, shopping, food preparation, housekeeping, laundry, mode of transportation, responsibility for own medications, and ability to handle finances, assesses the activities that support an independent life. The Lawton IADL scale was scored as dependent/independent and was generally scored a full eight points. However, three subscales in the IADL questionnaire (food preparation, housekeeping, and laundry) were excluded from the analysis because most Japanese older men seldom do these houseworks. Thus, the maximum score was five points in this study. The PSMS consists of six items, such as toileting, feeding, dressing, grooming, ambulating, and bathing, and can evaluate the basic activities involving physical self-care. Complete independence is scored 1 point, and requiring assistance is scored 0 points. The overall scores ranged from 0 to 6. We used the Japanese version of the Zarit Burden Interview (J-ZBI) to measure caregiver burden [25, 26]. The J-ZBI consists of 22 questions, with a maximum score of 88 points. A higher score indicated a greater burden.

### Statistical analyses

The demographic and clinical characteristics between groups were analyzed using the Kruskal-Wallis test or the chi-square test, given the skewed distribution of data. The significance level was set at *p* < 0.05. We compared the NPI total score, the SRI total score, and each subscale score of the NPI and SRI among groups using the Kruskal-Wallis test. If significant differences were found, a post hoc Dunn test was used. For multiple comparisons, the statistical threshold was set at *p* < 0.05/17 = 0.0029. Initially, bringing all SD patients together, each variable was compared among the three groups (bvFTD, SD-all, and AD) and then among the four groups (bvFTD, SD-R, SD-L, and AD) dividing SD patients into two groups. Additionally, we conducted a multiple regression analysis to clarify the clinical factors related to caregiver burden in each FTD group (bvFTD, SD-R, and SD-L). The following variables were entered as independent variables: MMSE score, NPI total score, SRI total score, PSMS score, and Lawton IADL scale score. The best models were derived using a stepwise regression analysis. The significance level was set at *p* < 0.05. All statistical analyses were performed using SPSS version 25.0 (IBM SPSS Japan, Tokyo, Japan).

## Results

**Fig. 1:**
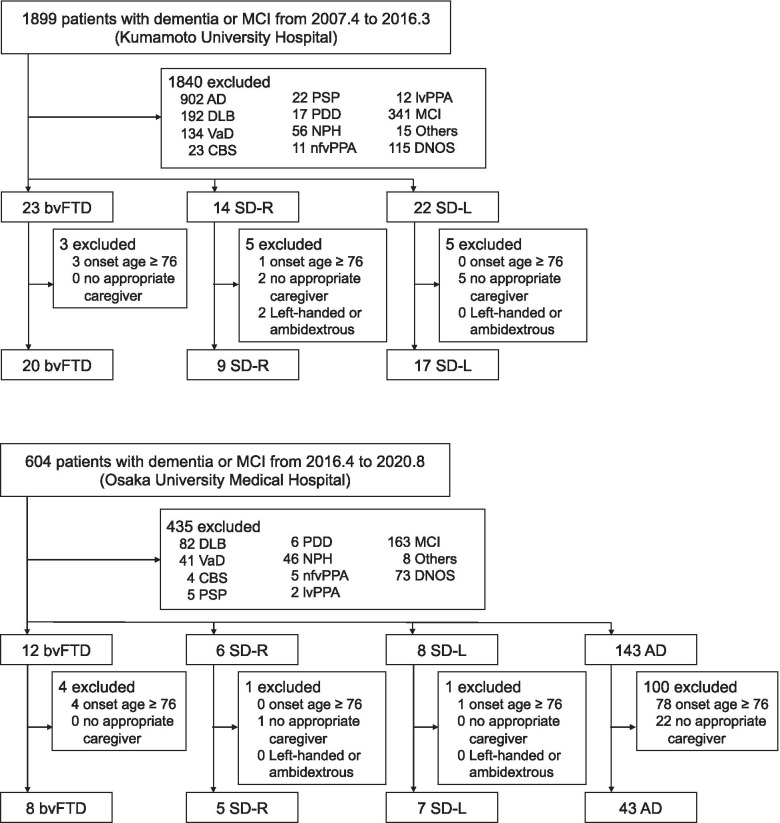
Participant selection. MCI, mild cognitive impairment; AD, Alzheimer’s disease; DLB, dementia with Lewy bodies; VaD, vascular dementia; CBS, corticobasal syndrome; PSP, progressive supranuclear palsy; PDD, Parkinson disease with dementia; NPH, normal pressure hydrocephalus; nfvPPA, non-fluent/agrammatic variant primary progressive aphasia; lvPPA, logopenic variant primary progressive aphasia; DNOS, dementia not otherwise specified; bvFTD, behavioral variant frontotemporal dementia; SD-R, right-sided predominant semantic dementia; SD-L, left-sided predominant semantic dementia

Of the 2503 patients who were diagnosed with dementia or mild cognitive impairment in the two institutes, 28, 14, 24, and 43 patients with bvFTD, SD-R, SD-L, and AD, respectively, met the inclusion criteria (Fig. 1). Table 1 shows the demographic details of each group. Patients in the AD groups were older than those in the bvFTD group. There were more females in the AD group than in the bvFTD group. The SD-L and AD groups had a significantly lower MMSE score than the SD-R group. There was no significant difference among the groups in terms of CDR. Regarding PSMS, the bvFTD group scored lower than the SD-all and AD groups among the three groups (bvFTD, SD-all, and AD) and scored lower than the SD-L and AD groups among the four groups (bvFTD, SD-R, SD-L, and AD). Regarding IADL, the bvFTD and AD groups scored lower than the SD-all group among the three groups and scored lower than the SD-L group among the four groups.

**Table 1:** Patient demographics and clinical characteristics

	AD group (*n* = 43)	bvFTD group (*n* = 28)	SD-all group (*n* = 38)	Comparison among AD, bvFTD, and SD-all groups			SD-R group (*n* = 14)	SD-L group (*n* = 24)	Comparison among AD, bvFTD, SD-R, and SD-L groups		
				*H*/*χ*^2^	*p*	Post hoc test			*H*/*χ*^2^	*p*	Post hoc test
Age, years	69.0 (8.5)	63.9 (7.2)	68.1 (6.5)	8.60	0.014^a^	AD > bvFTD	65.7 (5.7)	69.5 (6.7)	11.33	0.010^a^	AD > bvFTD
Age of onset, years	65.9 (7.8)	61.4 (7.6)	64.6 (6.9)	6.87	0.032^a^	AD > bvFTD	61.4 (6.9)	66.5 (6.2)	11.01	0.012^a^	Not significant
Sex (female)	30 (69.8%)	10 (35.8%)	23 (60.5%)	8.24	0.016^b^	AD > bvFTD	8 (57.1%)	15 (62.5%)	8.34	0.039^b^	AD > bvFTD
MMSE (/30)	19.4 (5.5)	18.7 (7.5)	19.4 (7.7)	0.63	0.729^a^	na	24.0 (3.8)	16.7 (8.1)	11.82	0.008^a^	SD-R > SD-L, AD
CDR severity	0.8 (0.6)	1.1 (0.8)	0.8 (0.5)	5.10	0.078^a^	na	0.9 (0.5)	0.7 (0.4)	6.63	0.085^a^	na
PSMS score (/6)	5.5 (1.2)	4.4 (1.9)	5.6 (1.1)	15.58	<0.001^a^	SD-all, AD > bvFTD	5.5 (0.9)	5.6 (1.2)	16.30	<0.001^a^	SD-L, AD > bvFTD
Lawton IADL score (/5)	3.7 (1.1)	3.0 (1.7)	4.5 (0.9)	18.84	<0.001^a^	SD-all > bvFTD, AD	4.3 (1.1)	4.6 (0.7)	19.44	<0.001^a^	SD-L > bvFTD, AD
J-ZBI score (/88)	20.0 (13.6)	30.8 (17.2)	17.3 (14.2)	13.30	0.0013^a^	bvFTD > SD-all, AD	26.3 (17.0)	12.1 (9.3)	21.56	<0.001^a^	bvFTD > SD-L, AD SD-R > SD-L

Values are expressed as *n* or mean (standard deviation). ^a^Kruskal-Wallis test. ^b^Chi-square

*Abbreviations*: *AD*, Alzheimer’s disease; *bvFTD*, behavioral variant frontotemporal dementia; *SD*, semantic dementia; *SD-R*, right-sided predominant semantic dementia; *SD-L*, left-sided predominant semantic dementia; *MMSE*, Mini-Mental State Examination; *CDR*, Clinical Dementia Rating; *PSMS*, Physical Self-Maintenance Scale; *IADL*, Instrumental Activities of Daily Living; *J-ZBI*, Japanese version of the Zarit Burden Interview; *na*, not available

**Fig. 2:**
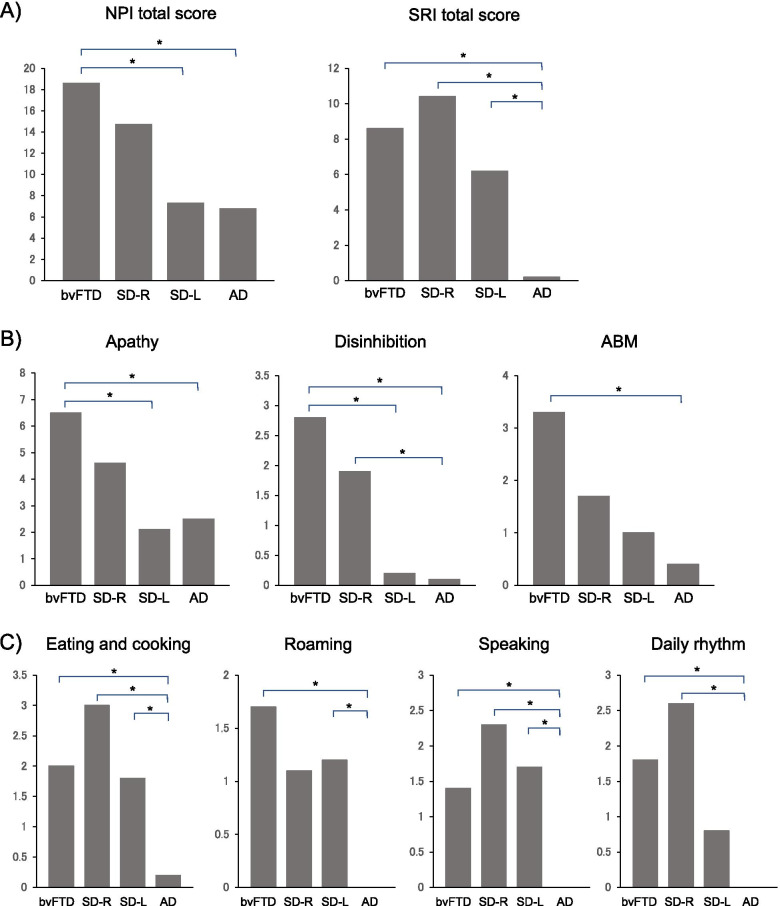
NPI and SRI scores among the bvFTD, SD-R, SD-L, and AD groups. **A** NPI total score and SRI total score. **B** NPI subscale scores. **C** SRI subscale scores. Data were analyzed using the Kruskal-Wallis test. ^*^p < 0.05/17 = 0.0029. NPI, Neuropsychiatric Inventory; SRI, Stereotypy Rating Inventory; bvFTD, behavioral variant frontotemporal dementia; SD-R, right-sided predominant semantic dementia; SD-L, left-sided predominant semantic dementia; AD, Alzheimer’s disease; AMB, aberrant motor behavior

The NPI and SRI results are shown in Table 2. Upon comparing the three groups (bvFTD, SD-all, and AD), significant differences were observed in the NPI total and NPI subscale scores of agitation, euphoria, apathy, disinhibition, and AMB. Post hoc tests revealed that the bvFTD group had significantly higher NPI total score (*H* = 20.63, *p* < 0.001) and NPI subscale scores of euphoria (*H* = 13.54, *p* = 0.0011), apathy (*H* = 20.44, *p* < 0.001), and AMB (*H* = 17.57, *p* < 0.001) than the SD-all and AD groups and that the bvFTD group had significantly higher NPI subscale scores of agitation (*H* = 11.70, *p* = 0.0029) and disinhibition (*H* = 16.67, *p* < 0.001) than the AD group. Upon comparing the four groups (bvFTD, SD-R, SD-L, and AD groups), the NPI total score (*H* = 24.99, *p* < 0.001) and NPI subscale scores of apathy (*H* = 24.78, *p* < 0.001) were significantly higher in the bvFTD group than in the SD-L and AD groups. NPI subscale score of disinhibition was significantly higher in the bvFTD group than in the SD-L and AD groups and significantly higher in the SD-R group than in the AD group (*H* = 20.78, *p* < 0.001). NPI subscale score of AMB was significantly higher in the bvFTD group than in the AD group (*H* = 17.61, *p* < 0.001). However, there were no significant differences between the bvFTD and SD-R groups for all NPI items. Upon comparing the three groups (bvFTD, SD-all, and AD), the bvFTD and SD-all groups had a significantly higher SRI total score (*H* = 40.90, *p* < 0.001) and SRI subscale scores of eating and cooking (*H* = 22.79, *p* < 0.001), roaming (*H* = 14.19, *p* < 0.001), speaking (*H* = 19.89, *p* < 0.001), and daily rhythm (*H* = 15.96, *p* < 0.001) than the AD group. SRI subscale score of movements was significantly higher in the bvFTD group than in the AD group (*H* = 13.00, *p* = 0.002). Upon comparing the four groups (bvFTD, SD-R, SD-L, and AD groups), the SRI total score (*H* = 42.75, *p* < 0.001) and the SRI subscale score of eating and cooking (*H* = 23.76, *p* < 0.001) and speaking (*H* = 21.31, *p* < 0.001) were significantly higher in the bvFTD, SD-R, and SD-L groups than in the AD group. The subscale score of roaming was significantly higher in the bvFTD and SD-L groups than in the AD group (*H* = 14.23, *p* = 0.0026). The daily rhythm score was significantly higher in the bvFTD and SD-R groups than in the AD group (*H* = 18.69, *p* < 0.001). Figure 2 shows the significant group differences among the four groups in the NPI and SRI items using the Kruskal-Wallis test.

**Table 2:** NPI score and SRI score across three groups (AD, bvFTD, and SD-all) or four groups (AD, bvFTD, SD-R, and SD-L)

	AD group (*n* = 43)	bvFTD group (*n* = 28)	SD-all group (*n* = 38)	Comparison among AD, bvFTD, and SD-all groups			SD-R group (*n* = 14)	SD-L group (*n* = 24)	Comparison among AD, bvFTD, SD-R, and SD-L groups		
				*H*/*χ*^2^	*p*	Post hoc test			*H*/*χ*^2^	*p*	Post hoc test
NPI total score	6.8 (7.2)	18.6 (12.3)	10.1 (11.4)	20.63	<0.001^*^	bvFTD > SD, AD	14.7 (13.6)	7.3 (9.1)	24.99	<0.001^*^	bvFTD > SD-L, AD
SRI total score	0.2 (1.0)	8.6 (7.7)	7.7 (9.5)	40.90	<0.001^*^	bvFTD, SD > AD	10.4 (10.6)	6.2 (8.6)	42.75	<0.001^*^	bvFTD, SD-R, SD-L > AD
NPI											
Delusions	0.5 (1.2)	0.0 (0.2)	0.1 (0.6)	10.62	0.05	na	0.3 (1.1)	0.0 (0.0)	11.16	0.01	na
Hallucinations	0.3 (1.1)	0.0 (0.0)	0.1 (0.7)	2.81	0.25	na	0.3 (1.1)	0.0 (0.2)	2.99	0.39	na
Agitation	0.2 (0.6)	1.6 (2.3)	1.0 (2.5)	11.70	0.0029^*^	bvFTD > AD	1.9 (3.7)	0.5 (1.3)	12.32	0.006	na
Depression	1.3 (2.2)	0.7 (1.9)	1.0 (2.4)	3.18	0.20	na	0.9 (1.3)	1.0 (2.9)	3.84	0.28	na
Anxiety	0.8 (2.2)	1.6 (3.4)	0.9 (2.5)	1.31	0.52	na	1.6 (3.8)	0.5 (1.2)	1.52	0.68	na
Euphoria	0.1 (0.6)	1.0 (1.9)	0.4 (2.0)	13.54	0.0011	bvFTD > SD, AD	0.9 (3.2)	0.2 (0.8)	13.66	0.0034	na
Apathy	2.5 (2.2)	6.5 (3.6)	3.0 (3.7)	20.44	<0.001^*^	bvFTD > SD, AD	4.6 (4.3)	2.1 (3.0)	24.78	<0.001^*^	bvFTD > SD-L, AD
Disinhibition	0.1 (0.5)	2.8 (3.9)	0.8 (1.9)	16.67	<0.001^*^	bvFTD > AD	1.9 (2.9)	0.2 (0.4)	20.78	<0.001^*^	bvFTD > SD-L, AD SD-R > AD
Irritability	0.6 (1.4)	1.1 (2.4)	1.4 (2.7)	3.49	0.18	na	0.9 (1.4)	1.8 (3.2)	3.76	0.29	na
AMB	0.4 (2.0)	3.3 (4.4)	1.3 (2.9)	17.57	<0.001^*^	bvFTD > SD, AD	1.7 (3.8)	1.0 (2.4)	17.61	<0.001^*^	bvFTD > AD
SRI											
Eating and cooking	0.2 (0.8)	2.0 (3.0)	2.3 (2.8)	22.79	<0.001^*^	bvFTD, SD > AD	3.0 (3.1)	1.8 (2.6)	23.76	<0.001^*^	bvFTD, SD-R, SD-L > AD
Roaming	0.0 (0.3)	1.7 (3.1)	1.2 (2.2)	14.19	<0.001^*^	bvFTD, SD > AD	1.1 (2.0)	1.2 (2.3)	14.23	0.0026^*^	bvFTD, SD-L > AD
Speaking	0.0 (0.0)	1.4 (2.5)	1.9 (3.3)	19.89	<0.001^*^	bvFTD, SD > AD	2.3 (3.4)	1.7 (3.3)	21.31	<0.001^*^	bvFTD, SD-R, SD-L > AD
Movements	0.0 (0.0)	1.6 (2.9)	0.8 (2.6)	13.00	0.002^*^	bvFTD > AD	1.0 (3.2)	0.7 (2.3)	13.03	0.005	na
Daily rhythm	0.0 (0.0)	1.8 (2.8)	1.4 (3.0)	15.96	<0.001^*^	bvFTD, SD > AD	2.6 (4.1)	0.8 (2.0)	18.69	<0.001^*^	bvFTD, SD-R > AD

Values are presented as mean (standard deviation). *NPI*, Neuropsychiatric Inventory; *SRI*, Stereotypy Rating Inventory; *AD*, Alzheimer’s disease; *bvFTD*, behavioral variant frontotemporal dementia; *SD*, semantic dementia; *SD-R*, right-sided predominant semantic dementia; *SD-L*, left-sided predominant semantic dementia; *AMB*, aberrant motor behavior; *na*, not available; ^*^p < 0.05/17 = 0.0029

The J-ZBI score was significantly higher in the bvFTD group than the SD-L and AD groups and significantly higher in the SD-R group than in the SD-L group (*H* = 21.56, *p* < 0.001) (Table 1). Multiple regression analysis revealed that the NPI total score (*β* = 0.558, *p* = 0.002) was significantly associated with the J-ZBI score in the bvFTD group (Table 3). In the SD-R group, the NPI total score (*β* = 0.567, *p* = 0.009) and the Lawton IADL scale score (*β* = −0.426, *p* = 0.037) were independently associated with the J-ZBI score. In the SD-L group, the NPI total score (*β* = 0.520, *p* = 0.004) and MMSE score (*β* = −0.351, *p* = 0.041) were independently associated with caregiver burden.

**Table 3:** Results of the multiple regression analysis of predictors of the J-ZBI score

**bvFTD**	***β***	***t***	**95% CI**	***p***
NPI total score	0.558	3.426	0.31; 1.25	0.002^*^
Adjusted *R*^2^ = 0.285				
**SD-R**	***β***	***t***	**95% CI**	***P***
NPI total score	0.567	3.150	0.21; 1.20	0.009^*^
Lawton IADL score	−0.426	−2.370	−12.28; −0.46	0.037^*^
Adjusted *R*^2^ = 0.656				
**SD-L**	***β***	***t***	**95% CI**	***P***
NPI total score	0.520	3.217	0.19; 0.87	0.004^*^
MMSE	−0.351	−2.176	−0.79; −0.02	0.041^*^
Adjusted *R*^2^ = 0.445				

*J-ZBI*, Japanese version of the Zarit Burden Interview; *bvFTD*, behavioral variant frontotemporal dementia; *SD-R*, right-sided predominant semantic dementia; *SD-L*, left-sided predominant semantic dementia; *NPI*, Neuropsychiatric Inventory; *IADL*, Instrument Activities of Daily Living; *MMSE*, Mini-Mental State Examination; ^*^p < 0.05

## Discussion

When SD was analyzed as one group, the NPI total score and NPI subscale scores for euphoria, apathy, and AMB were significantly lower than those of the bvFTD group, but comparable to those of the AD group. This result was consistent with that of Yiannopoulou et al. [9], which showed that patients with SD had milder neuropsychiatric symptoms than those with bvFTD. Upon dividing SD into the SD-R and SD-L groups based on temporal lobe atrophy predominance, we found no significant differences in the total NPI score and NPI subscale scores of disinhibition and apathy between the bvFTD and SD-R groups, although the bvFTD group had significantly higher scores than the SD-L group. The SD-R group scores were closer to those of the bvFTD group than the SD-L group. Recently, Ulugut Erkoyun et al. [5] reported that the prevalence of disinhibition and apathy at the first assessment in the rtvFTD group was higher than that in the svPPA group and lower than that in the bvFTD group. Since rtvFTD and svPPA are almost equivalent to our SD-R and SD-L, respectively, these results suggest that patients with SD show different behavioral profiles depending on the predominantly atrophic side and that right-sided predominant SD presents with similar conditions as bvFTD than left-sided predominant SD, at least in the early stage of the disease.

The neural mechanisms underlying the neuropsychiatric symptoms of SD remain unknown. In contrast, several studies have reported lesions responsible for behavioral symptoms in bvFTD. O’Connor et al. [27] reported that disinhibition was associated with specific atrophy of the right middle temporal region in bvFTD. Zomboni et al. [28] showed that the severity of disinhibition correlated with atrophy in the right nucleus accumbens, right superior temporal sulcus, and right mediotemporal limbic structures and that the severity of apathy correlated with atrophy in the right dorsolateral prefrontal cortex in bvFTD. Garcia et al. [29] reported that patients with disinhibition-debut showed atrophy in the right mediotemporal limbic structures and that the severity of apathy in apathetic-debut patients correlated with atrophy in the right dorsolateral prefrontal cortex and right insula in bvFTD. These studies emphasized the involvement of both frontal and temporal lobes, especially on the right side, in the development of disinhibition and apathy in patients with bvFTD. Ulugut Erkoyun et al. [5] reported that rtvFTD had predominant anterior temporal atrophy with a great degree on the right side and the ipsilateral ventral frontal areas, representing a close mirror image of svPPA. The relationship between bvFTD, SD-R, and SD-L revealed in our study reinforces the involvement of the right frontotemporal lobe in the behavioral symptoms of FTD. Moreover, previous studies suggested that SD-R might have a different pathological background from SD-L based on heredity. It has been pointed out that rtvFTD is not genetically sporadic [30, 31], while SD-L is a sporadic and pure transactive response DNA-binding protein 43 disorder. Although all the present patients with SD had no family history of SD, differences in background pathology as well as differences in lesions might influence the differences in neuropsychiatric symptoms between SD-R and SD-L.

Stereotypic behaviors are common symptoms of bvFTD and were adopted by the international consensus criteria as core symptoms for bvFTD [2]. Stereotypic behaviors range from simple repetitive movements to complex and compulsive behaviors. Few studies have compared the characteristics of stereotypic behaviors between bvFTD and SD, reporting that bvFTD and SD showed a similar prevalence of stereotypic behaviors [6]. In this study, SRI total score in the SD-all group was comparable to that in the bvFTD group and significantly higher than that in the AD group, which was consistent with previous studies [6]. However, when analyzing SD-R and SD-L separately, the SD-R group showed the highest SRI total score and three SRI subscale scores (eating and cooking, speaking, and daily rhythm) among the three FTD groups, although the difference was not statistically significant. Moreover, the SD-R group had a high score in more complex and compulsive items, such as eating and cooking and daily rhythm, rather than simple repetitive movements. Snowden et al. [13] pointed out that repetitive behaviors were common in both bvFTD and SD groups but had a more compulsive nature in the latter group. However, our results suggest that patients with SD-R, with the right side and temporal lobe pathology rather than the left side and frontal lobe pathology, showed the most complex and compulsive symptoms. Josephs et al. [14] reported that patients with bvFTD with stereotypies had greater striatum volume loss compared to those without stereotypies and that the loss was more severe on the right side and extended laterally to the right insula. Given that the insula and striatum are significantly atrophied in SD [32], it is reasonable that the SD-R group exhibited marked complex compulsive-like stereotypic behaviors.

Compared with non-caregivers, caregivers of patients with dementia have higher rates of depressive and anxiety disorders [33–35], lower quality of life [36], and greater mortality [37]. In this study, caregivers of patients with bvFTD felt a higher burden than those of patients with SD-L and AD, which was consistent with previous studies [15–17, 38]. It is noteworthy that caregivers of patients with SD-R felt a comparable burden to those of bvFTD and a higher burden than those of SD-L. Koyama et al. [38] reported that caregiver burden in SD-R was higher than that in SD-L, but the difference was not statistically significant. To our knowledge, this is the first study to clarify the degree of caregiver burden for SD-R. Furthermore, different factors increased the caregiver burden in each FTD subgroup, suggesting that FTD requires different approaches to reduce the burden on caregivers for each subgroup. In the SD-L group, the MMSE and NPI scores were significantly associated with caregiver burden. The MMSE score in the SD-L group was the lowest among the four groups, which is thought to reflect a remarkable language disturbance from the early stage of the disease. Thus, in SD-L, interventions for both speech and behavioral disorders are important. On the other hand, in the SD-R group, the Lawton IADL scale score was significantly related to the J-ZBI score independently of the NPI score, suggesting that attention should be paid to IADL disability as well as behavioral disorders from the viewpoint of caregiver burden in SD-R. Few reports have examined IADL in FTD, indicating that SD has less IADL dysfunction than bvFTD [39]. In this study, IADL disability was milder in the SD-R group than in the bvFTD group, similar to the previous study. Nevertheless, IADL disability was associated with caregiver burden in the SD-R group rather than the bvFTD group. Further research on the characteristics of IADL dysfunction and its impact on caregiver burden in SD-R may clarify this issue.

The strength of this study is that neuropsychiatric symptoms of FTD were assessed using established scales, such as the NPI and SRI. In addition, we recruited only right-handed patients with SD, which clarified the relationship between the dominant atrophic side and behavioral disorders.

### Limitations

There are some limitations that need to be addressed. First, the diagnosis relied solely on a clinical basis without histopathologic and genetic confirmation, with some uncertainty about the rate of misclassification. Second, the sample size of the SD-R was relatively small (*n* = 14), which may have caused a statistical type 2 error. Third, this was a cross-sectional study at the first visit, restricting some of our interpretations. Fourth, it is well known that the age of onset among bvFTD, SD, and AD varies [40, 41]. In addition, there are some differences in clinical profiles between early-onset and late-onset subgroups in bvFTD, SD, and AD [42–44]. Therefore, we only included patients with onset before the age of 76 years to match the age of onset among the groups as much as possible. Careful judgment is required when applying the present results to older patients with FTD.

## Conclusions

Patients with SD showed different behavioral profiles depending on the predominantly atrophic side, and SD-R seems to be a similar condition to bvFTD rather than SD-L in terms of behavioral disorders. Caregiver burden for SD-R was comparable to that of bvFTD and higher than that of SD-L patients. Different factors influenced the increased caregiver burden among bvFTD, SD-R, and SD-L. Overall, the results of this study suggest that it is important to distinguish SD into SD-R and SD-L and take different interventional approaches for FTD among bvFTD, SD-R, and SD-L.

## Data Availability

Research data are not shared.

## Abbreviations

FTD: Frontotemporal dementia
bvFTD: Behavioral variant frontotemporal dementia
SD: Semantic dementia
PNFA: Progressive non-fluent aphasia
PPA: Primary progressive aphasia
svPPA: Semantic variant primary progressive aphasia
NPI: Neuropsychiatric Inventory
SD-L: Left-sided predominant semantic dementia
SD-R: Right-sided predominant semantic dementia
rtvFTD: Right temporal variant of frontotemporal dementia
AD: Alzheimer’s disease
MRI: Magnetic resonance imaging
AMB: Aberrant motor behavior
SRI: Stereotypy Rating Inventory
MMSE: Mini-Mental State Examination
CDR: Clinical Dementia Rating
ADL: Activities of Daily Living
IADL: Instrument Activities of Daily Living
PSMS: Physical Self-Maintenance Scale
J-ZBI: Japanese version of the Zarit Burden Interview
MCI: Mild cognitive impairment
DLB: Dementia with Lewy bodies
VaD: Vascular dementia
CBS: Corticobasal syndrome
PSP: Progressive supranuclear palsy
PDD: Parkinson disease with dementia
NPH: Normal pressure hydrocephalus
nfvPPA: Non-fluent/agrammatic variant primary progressive aphasia
lvPPA: Logopenic variant primary progressive aphasia
DNOS: Dementia not otherwise specified
